# Do Patients with Chronic Spinal Pain and Comorbid Insomnia Have More Features of Central Sensitization? A Case-Control Study

**DOI:** 10.3390/healthcare11243152

**Published:** 2023-12-12

**Authors:** Lucas Araújo Almeida, Thomas Bilterys, Eveline Van Looveren, Olivier Mairesse, Barbara Cagnie, Mira Meeus, Maarten Moens, Dorien Goubert, Wouter Munneke, Lieven Danneels, Kelly Ickmans, Paula Rezende Camargo, Jo Nijs, Anneleen Malfliet, Liesbet De Baets

**Affiliations:** 1 Pain in Motion Research Group (PAIN), Department of Physiotherapy, Human Physiology and Anatomy (KIMA), Faculty of Physical Education & Physiotherapy, Vrije Universiteit Brussel, Laarbeeklaan 103—Building F, 1090 Brussel, Belgium; lucas.almeida@estudante.ufscar.br (L.A.A.); tbilter1@hfhs.org (T.B.); eveline.vanlooveren@komoptegenkanker.be (E.V.L.); wouter.munneke@vub.be (W.M.); kelly.ickmans@vub.be (K.I.); jo.nijs@vub.be (J.N.); anneleen.malfliet@vub.be (A.M.); 2 Laboratory of Analysis and Intervention of the Shoulder Complex, Department of Physical Therapy, Universidade Federal de São Carlos, São Carlos 13565-905, SP, Brazil; prcamargo@ufscar.br; 3 Department of Physical Therapy, University of Florida, Gainesville, FL 32611, USA; 4 Pain in Motion International Research Group, 1090 Brussel, Belgium; mira.meeus@uantwerpen.be (M.M.); maarten.ta.moens@vub.be (M.M.); dorien.goubert@imec.be (D.G.); 5 Department of Psychology, University of Warwick, Coventry CV4 7AL, UK; 6 Institute of Advanced Study, University of Warwick, Coventry CV4 7AL, UK; 7 Department of Rehabilitation Sciences and Physiotherapy, Faculty of Medicine and Health Sciences, Ghent University, Campus Heymans—Building B3, De Pintelaan 185, 9000 Ghent, Belgium; barbara.cagnie@ugent.be (B.C.); lieven.danneels@ugent.be (L.D.); 8 Brain, Body and Cognition (BBCO), Faculty of Psychology and Educational Sciences, Vrije Universiteit Brussel, 1090 Brussels, Belgium; olivier.mairesse@vub.be; 9 Department of Rehabilitation Sciences and Physiotherapy (MOVANT), Faculty of Medicine and Health Sciences, University of Antwerp, Campus Drie Eiken, Universiteitsplein 1, 2610 Wilrijk, Belgium; 10 Department of Neurosurgery and Radiology, University Hospital Brussels, 1090 Brussels, Belgium; 11 Stimulus Research Group, Vrije Universiteit Brussel, 1090 Brussels, Belgium; 12 Center of Neurosciences, Vrije Universiteit Brussel, 1090 Brussels, Belgium; 13 Department of Sport and Rehabilitation Sciences, University of Liège, 4000 Liege, Belgium; 14 Chronic Pain Rehabilitation, Department of Physical Medicine and Physiotherapy, University Hospital Brussels, 1090 Brussels, Belgium; 15 Movement & Nutrition for Health & Performance Research Group (MOVE), Department of Movement and Sport Sciences, Faculty of Physical Education and Physiotherapy, Vrije Universiteit Brussel, Pleinlaan 2, 1050 Brussels, Belgium; 16 Department of Health and Rehabilitation, Unit of Physiotherapy, Institute of Neuroscience and Physiology, Sahlgrenska Academy, University of Gothenburg, 41390 Göterbog, Sweden; 17 Research Foundation—Flanders (FWO), 1000 Brussels, Belgium

**Keywords:** central sensitization, chronic pain, chronic spinal pain, insomnia, polysomnography

## Abstract

Background: Chronic spinal pain (CSP) is a major public health problem worldwide, frequently related to sleep problems. Central sensitization (CS) may worsen the clinical picture of CSP patients with insomnia. The aim of this study was to compare self-reported and objectively measured clinical outcomes between insomniac CSP patients with comorbid insomnia with and without symptoms of CS. Methods: A case-control study on baseline self-reported sleep, functioning, and psychological distress through online questionnaires. Objective sleep and physical activity parameters and pressure pain thresholds (PPTs) were assessed through polysomnography, actigraphy, and digital algometry, respectively. Independent sample t-test and Mann–Whitney U tests were used to examine possible differences in the outcome measures between the groups. Results: Data from 123 participants were included and revealed no statistically significant group for objective sleep and physical activity parameters. The CS group, however, presented with worse self-reported sleep (quality sleep, insomnia severity, and dysfunctional beliefs about sleep), increased mental and physical fatigue, and higher psychological distress (anxiety and depressive symptoms), and reported lower PPTs. Conclusions: symptoms of CS may influence perceived sleep and affect functional health and well-being perception but do not seem to affect objective sleep and physical activity.

## 1. Introduction

Chronic spinal pain (CSP) is one of the most commonly reported conditions among people with chronic musculoskeletal pain [1,2,3]. It negatively affects personal health and overall well-being and contributes to a financial burden on the community and the health system [2,4,5]. CSP is frequently related to physical and mental comorbidities that may influence the patient’s functioning and treatment response [6,7]. Insomnia has long been reported as one of the most common and deteriorating comorbidities in people with CSP [8,9]. Insomnia may cause reduced sleep duration and quality, a significantly higher sleep onset latency, and self-perceived sleep dissatisfaction and sleep-related distress [9]. Furthermore, insomnia has been associated with worsened pain, mood, functioning [10,11,12], and inadequate pain inhibition [13], presenting a potential risk for the persistence of symptoms in CSP patients.

Central sensitization (CS) is defined as an increased response to neural signaling and a decrease in opioid receptor availability within the central nervous system, resulting in pain hypersensitivity [14,15,16]. It is well known that sleep disturbance associated with chronic pain facilitates the development of CS, leading to a reduced capacity of the central nervous system to inhibit pain, thereby facilitating chronic comorbid conditions [17,18]. However, the relationship between sleep disturbance and such sensory hypersensitivity remains unclear in CSP patients [17,18,19,20]. This knowledge gap may be explained by the influence of psychological factors on the clinical picture of the CSP condition [21,22,23,24,25], and also by the high variability of CS symptoms in patients who suffer from CSP [26]. Anxiety, depressive symptoms, and fatigue seem to modulate insomnia severity and hypersensitivity in a subgroup of CSP patients [27]. Thus, identifying subgroups of insomniac CSP patients may be clinically relevant to guide clinicians to a better understanding of symptom variability and treatment response.

Given the available evidence regarding the relationship between pain hypersensitivity, insomnia, and psychological aspects in the general population and in people with chronic musculoskeletal pain conditions, CS may contribute to insomnia severity and the physical/mental functioning of CSP patients. Therefore, this study aimed to examine the difference in sleep, physical/mental functioning, and psychological distress in patients with CSP associated with comorbid insomnia and presenting hypersensitivity symptoms. We hypothesized that insomniac CSP patients in the CS group would present worse results compared to the non-CS group in all outcomes measured in this study.

## 2. Material and Methods

### 2.1. Study Design and Settings

This is a case-control study that examined possible differences in sleep features, physical activities, anxiety, depressive symptoms, fatigue, and pressure pain threshold between insomniac CSP patients with and without symptoms of CS. The study protocol was approved by the University Hospital Brussel and University Hospital Ghent Ethics Committees. This study had its protocol registered at clinicaltrials.gov (no. NCT03482856) and published elsewhere [28]. All participants included were informed about the study procedures and signed informed consent before study enrolment.

### 2.2. Participants

The sample size was estimated specifically for this case-control study. The sample size calculation was performed with G*Power 3 (Düsseldorf, Germany) and it was calculated for a comparison analysis based on a medium effect size of 0.565, which was estimated based on a pilot study by Jungquist et al. that used the Insomnia Severity Index to assess insomnia severity [29]. The calculation was based on two-tailed testing (alpha = 0.05; aiming for 95% power), and an allocation ratio (N2/N1) of one. The sample size calculation resulted in a total of 102 participants (n = 51 in each group). One hundred and forty-six potential participants with CSP and comorbid insomnia were recruited through different sources: health institutions, advertisements in social media and printed newspapers, and patient support groups. Potential participants received written information about the study and were requested to complete questionnaires remotely to screen for inclusion and exclusion. Inclusion and exclusion criteria are described in Table 1. At the end, 123 participants were included in this study.

### 2.3. Outcomes Measures

All outcomes were assessed in line with IMMPACT/OMERACT recommendations [30] and assessed at baseline. All assessments were performed by the same researchers (TB, WM) extensively trained by researchers (MM, OM, JN, AM) with broad experience in applying the outcome measures. This study used online questionnaires in Dutch through the REDCap platform to collect sociodemographic and pain-related information (including pain severity, pain interference in daily activities, pain duration, and pain location). Online questionnaires were also used to assess sleep, functioning, and psychological distress. Objective sleep, physical activity, and pressure pain threshold (PPT) measures were assessed using polysomnography, actigraphy, and algometry, respectively. In the information brochure of this study, which comes together with the informed consent, all potential participants were informed about the aims of the study and the related data collection. Here it is specified that both physical assessments and online questionnaires are used as outcomes in this study. An explanatory email along with the link to the questionnaires was composed and sent to the participants. Also, a phone number was added in this email where participants could get in contact with a researcher of this study in case of problems with filling out the digital questionnaires. We hypothesized that insomniac CSP patients in the CS group would present worse results compared to the non-CS group in all outcomes measured in this study.

#### 2.3.1. Pain-Related Information

Pain duration and pain location were addressed through a form developed by the researchers involved in this study. The Brief Pain Inventory (BPI) was used to address pain severity and pain interference in daily activities [31]. The BPI is a patient-reported outcome measure that provides a rate of pain intensity, and pain interference in functioning domains, such as mood, walking ability, interpersonal relationships, and ability to enjoy life [31]. All its items are rated via a numerical rating scale from 0 to 10, with 0 meaning “no pain”or “no interference”, and 10 corresponding to “pain as bad as you can imagine”or “interferes completely” [31]. 

#### 2.3.2. Sleep-Related Outcomes

All participants performed a one-night evaluation using home-based polysomnography (Alice PDX system, Philips Respironics Inc., Murrysville, PA, USA) [28] in the comfort of their own home to counteract potential (reversed) first-night effects. The polysomnography montage followed the American Academy of Sleep Medicine recommendations [32]. Participants were provided with both written and vocal instructions on the stages involved in the polysomnography montage by a trained researcher. Participants were also instructed to activate the event marker to indicate “lights off” and “lights on”. The polysomnography assessment provides the following parameters: time in bed, total sleep time, sleep onset latency, wake duration after sleep onset, and sleep efficiency [28]. Polysomnography is considered the “gold standard” for monitoring sleep [33].

Self-reported sleep outcomes were addressed using the Pittsburg Sleep Quality Index (PSQI) [34], the Insomnia Severity Index (ISI) [35,36], the Dysfunctional Beliefs and Attitudes about Sleep Scale (DBAS-16) [37], and the Epworth Sleepiness Scale (ESS) to assess sleep propensity [38]. The PSQI is a questionnaire to assess subjective sleep quality and contains 19 statements regarding sleep latency, sleep duration, habitual sleep efficiency, sleep disturbances, and use of sleeping medication [39]. PSQI scores range from 0 to 21, with higher scores indicating worse sleep disturbance [34]. The ISI is a questionnaire that contains seven items assessing the concerns related to insomnia [40], such as severity of sleep onset, sleep maintenance difficulties, and satisfaction with current sleep pattern [35]. Each item is rated on a 0–4 scale; the total score ranges from 0 to 28, and a higher score indicates worse insomnia severity [41]. DBAS-16 is a brief questionnaire used to assess patients’ sleep-disruptive cognitions [35,37]. The 16 statements are rated on a 0–10 Likert scale [42] and the scores range from 0 to 160. Higher scores indicate greater dysfunctional beliefs about sleep [37]. The Epworth Sleepiness Scale (ESS) measures the chances of falling asleep in eight different daily life situations [43]. The total ESS score ranges from 0 to 24, and a higher score reflects a greater sleepiness level [43].

#### 2.3.3. Physical Activity and Functioning Outcome

The physical activity level was evaluated using actigraphy [44]. Three-axis accelerometer activity monitors (GT9X-BT, Actigraph Corporation, LLC, Pensacola, FL, USA) assessed the physical activity level for 7 consecutive days. Participants were instructed to continuously wear the activity monitors (day and night) on their nondominant wrist. ActiLife6 (Actigraph Corporation, LLC) was used to analyze the data captured with the activity monitors, and average values of physical activity were used in the statistical analyses [45]. Actigraph devices are commonly used in research and validated for the general population [45]. The Short Form Health Survey-36 (SF-36) [46] was used to address physical and mental domains related to self-reported quality of life [46] and is scored on a scale from 0 to 400, with 400 indicating the best functioning level [46].

#### 2.3.4. Anxiety, Depressive Symptoms, and Fatigue

The Hospital Anxiety and Depression Scale (HADS) assesses affective symptoms like anxiety and depression [47] in 14 statements (7 to address anxiety and 7 to measure depressive symptoms) [47] scored on a numeric rating scale from 0 to 4 points [47]. The total score of each subscale is calculated separately, with scores ranging from 0 to 21 points, and higher scores indicate worse anxiety and depressive symptoms [47]. The Brugmann Fatigue Scale (BFS) is a questionnaire with psychometric measurements to assess subjective fatigue levels through assessing mental and physical rest propensity [48]. BFS contains eight statements (four statements to assess mental fatigue and four statements addressing physical fatigue) [48], which are rated on a Likert scale (0 = “It’s very unlikely that I need to rest” to 3 = “it’s almost sure that I need to rest”) [48]. Higher scores indicate higher rest propensity [48].

#### 2.3.5. Pressure Pain Thresholds

The pressure pain threshold (PPT) test determines the amount of nonpainful pressure stimulus that turns into a painful sensation [49,50,51]. PPTs were evaluated using a hand-held electronic pressure algometer (Wagner instruments) applied at a local (symptomatic) site and a remote (asymptomatic) site. For patients with cervical pain, the trapezius muscle was considered as a local site and the calf was evaluated as a remote site. Regarding low back pain patients, the lumbar paravertebral muscle and web between the thumb and the index were considered as local and remote sites, respectively [28]. The order of test sites was randomized, and the result was defined by the mean of two measurements [28].

### 2.4. Procedure

Participants for the study were enrolled through a process that involved assessing patient eligibility among those seeking care and obtaining their Informed Consent. Subsequently, baseline assessments of the outcomes were performed. Participants were called via telephone to schedule the home-based polysomnography assessment to screen for underlying sleep pathologies, and to deliver the wristwatch to register rest and activity cycles.

Group categorization was determined based on the total score from the Central Sensitization Inventory (CSI). The CSI serves as a screening instrument for identifying overlapping symptoms of CS and ascertaining whether a patient’s symptoms might be associated with CS [52]. Scores higher than 40 indicate the presence of symptoms of CS, and it was used to split the groups into the presence or absence of self-reported symptoms of CS [15]. CSI consists of 25 items assessing health-related symptoms rated on a Likert scale (0 = “never” to 4 = “always”) [14]. The scores range from 0 to 100, representing the degree of self-reported symptomatology [14]. CSI has proven psychometric strength [53].

### 2.5. Statistical Analysis

Statistical analyses were performed with SPSS 26.0 (IBM, Armonk, NY, USA). Descriptive statistics were performed for all demographic data and outcome measures. Continuous variables were expressed as means and standard deviations, and the normality assumptions were checked using histograms, Q-Q plots, and Kolmogorov–Smirnov tests. Pearson’s chi-square test was used to compare demographic categorical variables between groups. Independent sample t-test and Mann–Whitney U tests were used to compare the outcome measures between CSP patients with and without symptoms of CS based on the CSI (referred to as CS group and non-CS group, respectively). Statistically significant differences were defined at alpha 0.05 [54].

## 3. Results

**Figure 1:**
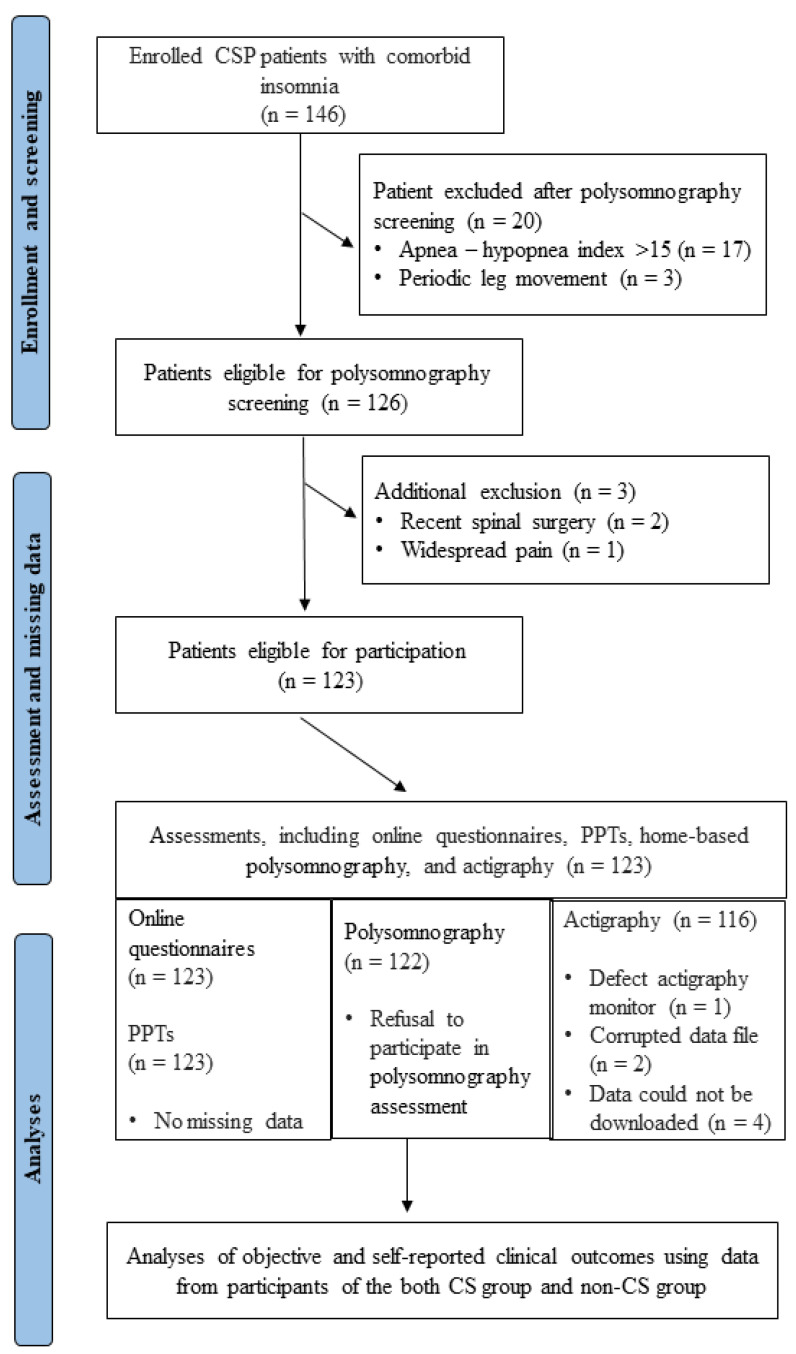
Flowchart of the cross-sectional study. CSP = chronic spinal pain; CS = central sensitization; PPT = pressure pain thresholds.

Data from 123 participants were analyzed. A detailed overview of enrollment, screening, and measurement is presented in Figure 1. Demographic data and a description of pain location are presented in Table 2. The mean ± standard deviation of pain severity (scores ranging from 0 to 10) was 4.5 ± 1.5 and 4.1 ± 1.4 in the CS group and non-CSI group, respectively. In addition, pain interference in daily activities in the CS group was 3.4 ± 1.9, while in the non-CS group it was 2.5 ± 1.3. Furthermore, neither group performed vigorous physical activities during the 7 days measured.

### 3.1. Comparison Outcomes between CS and Non-CS Group

#### Sleep Outcomes

During the polysomnography, one participant from the CS group refused to participate in the polysomnography assessment. We did not observe significant statistical differences between the groups for time in bed (U = 1568.000; *p* = 0.6); sleep onset latency (U = 1575.000 *p* = 0.7); and sleep efficiency (U = 1376.000; *p* = 0.15). However, we did observe statistical differences between the groups for total sleep time (U = 1312.000; *p* = 0.07), and wake duration after sleep onset (U = 1323.500; *p* = 0.08), although it was not significant at a 95% level in this study. Furthermore, there was no statistical difference between the groups for sleepiness (*p* = 0.30). On the other hand, the CS group presented worse self-reported sleep outcomes, such as sleep quality, insomnia severity, and dysfunctional beliefs and attitudes about sleep, compared to the non-CS group (Table 3).

### 3.2. Physical Activity and Functioning Outcome

The actigraphy analysis has missing data due to a defect in the actigraphy monitor (n = 1), a corrupted actigraphy data file (n = 2), and a failure to download the actigraphy data file (n = 4). Results of objective physical activity levels (sedentary, moderate, vigorous, and very vigorous) measured using actigraphy showed no significant differences between groups. In addition, no participant in both groups performed vigorous or very vigorous activities during their participation in this study. However, the CS group presented worse results regarding self-reported physical and mental functioning measured using SF-36 (*p* < 0.01). Physical activity and functioning results are presented in Table 4.

### 3.3. Anxiety, Depressive Symptoms, and Fatigue Outcomes

The CS group had worse anxiety (*p* < 0.001) and depressive symptoms (*p* = 0.02) in comparison to the non-CS group. There was a statistically significant difference between groups in the propensity for physical fatigue and the propensity for mental fatigue, with the CS group showing higher levels of fatigue (Table 5).

### 3.4. Pressure Pain Thresholds

The PPTs showed statistical differences between groups, with the CS group presenting lower PPTs on symptomatic and remote sites (Table 6).

## 4. Discussion

This study investigated the differences in sleep measures, physical activities, anxiety, depressive symptoms, fatigue, and pressure pain thresholds between insomniac CSP patients with and without symptoms of CS. The results of this study show that insomniac CSP patients with symptoms of CS presented worse self-reported sleep quality, higher insomnia severity, and more dysfunctional beliefs and attitudes about sleep compared to insomniac CSP patients without symptoms of CS. Furthermore, self-reported physical and mental functioning showed statistical differences between groups, with lower scores related to the CS group. These results indicate that symptoms of CS may affect self-reported functional health and well-being perception in CSP patients with insomnia. The CS group also presented worse anxiety, depressive symptoms, and fatigue (in physical and mental subscales).

Symptoms of CS did not influence objective sleep assessed using polysomnography, while they did affect self-reported sleep-related outcomes. Three possibilities may explain the discrepancies between self-reported and objective sleep parameters. First, we did observe a difference for total sleep time (*p* = 0.07) and duration of wakefulness after sleep onset (*p* = 0.08), but they were not significant at a 95% significance level. A larger sample size could present a significant difference in the statistical analysis for these outcomes. It is known that CS is a neural phenomenon that might lead to general and pain hypersensitivity [55] and that the CSI cannot directly assess CS [55]. In this study, the CSI was used to split the groups. This was done as the CSI is a clinically applicable tool, thereby providing clinically applicable results [14,16,56]. Our hypothesis that insomniac CSP patients in the CS group would present worse results compared to the non-CS group in self-reported and objective measures was based on evidence that showed an association between higher numbers of chronic comorbidities and more significant negative effects on functioning in patients with CSP [6,54]. However, the dichotomy of the CSI score does not consider patients with moderate symptoms of hypersensitivity. The descriptive analysis showed that about 25% of participants included in this study presented a score ranging between 30 and 39 points. According to Neblet and collaborators [56], scores higher than 30 points may be considered mild symptoms of CS [56] and this may have influenced the polysomnography results. However, this is an unlikely possibility because our cross-sectional data showed a significant difference between groups related to CSI scores. In other words, we expected patients with severe and extreme levels of CS would present longer time in bed, sleep onset latency, and wake duration after sleep onset, and shorter total sleep time, and lower sleep efficiency [57,58]. A second possibility is that CSP patients with comorbid insomnia generally underestimate self-reported total sleep time and overestimate time in bed and sleep onset latency [59]. Therefore, the discrepancy between self-reported and objective sleep measures suggests that CS severity may not affect the objective sleep measures but seems to influence perceived sleep.

Symptoms of CS did not influence physical activity levels, assessed using actigraphy. Physical activity is widely recognized for improving well-being and decreasing risks of chronic diseases [60,61] including musculoskeletal chronic pain [62]. A systematic review also suggests higher levels of regular physical activity as a protective factor for pain sensitivity in healthy individuals [63]. Considering the neurophysiological mechanisms, moderate to high physical activity levels may modulate the state of central pain inhibitory pathways and the immune system, resulting in a beneficial effect against perceived pain [64,65]. However, the relationship between regular physical activity and decreased pain sensitivity remains unclear in chronic pain patients [64]. Zoet et al. 2020 have synthesized evidence about the central neurobiological effects of physical exercise and reported very low-quality evidence that physical activity may exert effects on brain neurobiology in people with chronic pain [65]. Our results showed no significant difference in physical activity levels in insomniac CSP with and without symptoms of CS. According to the literature, there is no association between physical activity and the presence of insomnia in people with CSP [66,67]. Therefore, the fact that insomniac CSP patients with and without CS have the same physical activity level may be explained by pain influence and not whether there is insomnia or CS [68]. Interestingly, despite no group difference in objective physical activity measures, insomniac CSP patients with CS presented worse self-reported physical and mental functioning via SF-36. This result indicates that worse perceived sleep and higher psychological distress may play an important role in self-reported well-being results.

Psychological distress, such as anxiety, depressive symptoms, and fatigue are more prevalent in chronic pain patients than in the general population [69,70]. Furthermore, symptoms of CS seem to be an important mediator of the relationship among anxiety symptoms, depression symptoms, and pain intensity in chronic musculoskeletal pain [70]. Regarding the CSP condition, there is a strong relationship between severe CSI scores and higher levels of anxiety, depressive symptoms, and poor sleep quality [71]. The results of the present study reinforce the association between worse symptoms of CS and psychological distress [70,71]. The finding that self-reported sleep quality, higher insomnia severity, and more dysfunctional beliefs and attitudes about sleep are present in insomniac CSP patients with symptoms of CS suggests that this subgroup of the CSP population may benefit more from cognitive behavioral therapy for insomnia, the best evidence treatment for insomnia {Cheng, 2012 #8213} [29,72]. Indeed, cognitive behavioral therapy for insomnia specifically targets dysfunctional beliefs and attitudes about sleep [29,72]. Additionally, CS patients presented lower PPTs than non-CS patients, indicating an altered mechanical sensory response that is a sign of sensory hypersensitivity characteristics in CS [25,73,74]. 

This study presents limitations and strengths that must be considered when interpreting the results. First, as a cross-sectional study, we cannot make any claims about causation. Our results only reflect the clinical picture at the specific time of the evaluation. Second, the unbalanced number of women in both groups may have influenced the results, although sex differences in measures of central sensitization and pain sensitivity on experimental sleep disruption are currently not clear [31]. Third, the fact that the group was dichotomized based on a CSI cut-off should be highlighted. Future studies evaluating CS severity within CSP patients and comorbid insomnia are necessary to verify its impact on self-reported clinical outcomes. Fourth, the findings of this study should not be directly translated to chronic pain conditions other than chronic spinal pain, which is defined as chronic neck or low back pain in this study. Therefore, clinicians should be aware that these findings may not cover general dorsal column pain. Study strengths include the large sample size, the use of gold standard equipment for valid diagnosis of insomnia and sleep variables (i.e., the use of polysomnography), the blinding of the outcome assessor, and the improved understanding of the clinical picture of insomniac CSP patients and how CS may affect self-perceived clinical outcomes. Thus, this study can potentially encourage clinicians to address insomnia in assessing CSP patients. Furthermore, current findings can be helpful in identifying the characteristics of the subgroup of insomniac CSP patients that might experience or develop negative consequences related to central hypersensitivity.

## 5. Conclusions

The current results indicate that insomniac CSP patients with symptoms of central sensitization, defined by a score of 40 or more on the CSI, seem to present with poorer self-perceived sleep quality, and worse physical and mental functioning. In addition, the CS group presented higher levels of anxiety, depressive symptoms, fatigue, and lower PPTs. On the other hand, there was no significant difference between groups in objective sleep and physical activity measures. These results suggest that, within this sample, the presence of symptoms of CS may influence perceived but not objective sleep parameters, and affect functional health and well-being perception but not objectively measured physical activity.

## Figures and Tables

**Table 1:** Inclusion and exclusion criteria.

Inclusion	Exclusion
Aged between 18 and 65 years	Severe underlying sleep pathology (identified through baseline data of polysomnography)
Nonspecific spinal pain (≥3 months duration), and presence of pain ≥ 3 days/week.	Neuropathic pain and chronic widespread pain syndromes
Seeking care because of neck pain or low back pain	Shift workers
Native Dutch speaker	Being pregnant or became a parent in the preceding year
Having insomnia: no presence of other intrinsic sleep disorders	Thoracic pain in absence of neck or low back pain
Not starting new treatments or medication, and continuing their usual care six weeks prior to and during study participation (to obtain a steady state)	Spinal surgery history (i.e., surgery for spinal stenosis)
Refraining from analgesics and other substances that modulate the nervous system (caffeine, alcohol, or nicotine) in the 48 h prior to the assessments	Body Mass Index > 30 kg/m^2^
Nonspecific failed back surgery > 3 years are permitted	Current depression diagnosed by a doctor
Not undertaking exercise 3 days before the assessments	Not willing to refrain from analgesics in the 48 h prior to the assessments

**Table 2:** Demographics and pain-related information from both groups (n = 123).

Characteristics	CS Group (n = 82)	Non-CS Group (n = 41)	95% CI	*p*-Value
Age, years	38.7 ± 10.2	43.6 ± 12	0.80, 9.02	0.02
Body Mass Index, kg/m^2^	23 ± 3.3	23.9 ± 2.74	−0.32, 2.04	0.15
Pain duration, months	87 ± 92.3	93.4 ± 104	−30.7, 43.4	0.70
Sex, n (%)				
Female	64 (78)	18 (44)	−0.51, −0.17	<0.001
Pain location, n (%)				
Cervical pain	50 (61)	21 (51.2)	−0.42, 1.58	0.19
Back pain	32 (39)	20 (48.8)	−0.06, 0.31	0.21
CSI score range, n (%)				
0–29	−	10 (8)		
30–39	−	31 (25)		
40–49	45 (36)	−		
50–59	26 (21)	−		
60–100	11 (9)	−		
CSI total score	49.5 ± 7.4	32 ± 6	−19.9, −15.0	<0.001

Values are mean ± standard deviation unless otherwise indicated. CS = central sensitization; CSI = central sensitization index; n = sample size.

**Table 3:** Between-group differences in sleep outcomes.

**Outcomes**	**CS Group** **(n = 81)**	**Non-CS Group** **(n = 41)**	**95% CI**	***p*** **-Value**
Objective sleep, median (Q3–Q1)				
Time in bed	476.7 (95)	471.7 (82.1)	−36.3, 18.5	0.60
Total sleep time	433.5 (79.1)	409.5 (87)	−42, 3.3	0.07
Sleep onset latency	10.5 (12)	9.5 (12)	−8.8, 4.7	0.70
Wake duration after sleep onset	26.7 (25.5)	37.7 (49.5)	−0.53, 25.7	0.08
Sleep efficiency	91.4 (8.1)	90.7 (8.3)	−5.2, 0.37	0.15
Self-reported sleep, mean ± SD				
**Outcomes**	**CS group** **(n = 82)**	**Non-CS group** **(n = 41)**	**95% CI**	***p*** **-Value**
PSQI	10.1 ± 2.6	8.3 ± 2.3	−2.7, −0.90	<0.01
ISI	16.2 ± 3.8	13 ± 3.8	−0.73, −0.33	<0.01
DBAS-16	3.17 ± 0.4	2.65 ± 0.6	−0.61, −0.24	<0.01
ESS	8.5 ± 4.7	7.7 ± 4.5	−2.5, 0.96	0.30

CS = central sensitization; SD = standard deviation; CI = confidence interval; PSQI = Pittsburgh sleep quality index; ISI = insomnia severity index; DBAS-16 = dysfunctional beliefs and attitudes about sleep scale; ESS = Epworth sleepiness scale; Q = quartile; n = sample size.

**Table 4:** Between-group differences in physical activity and functioning outcomes.

Outcomes	CS Group (n = 76)	Non-CS Group (n = 40)	95% CI	*p*-Value
Actigraphy, mean ± SD				
% in light	39.3 ± 6.1	39 ± 5.4	−2.5, 1.96	0.79
% in sedentary	48.4 ± 7.2	50 ± 6.9	−1.1, 4.27	0.20
% in moderate	12.1 ± 4.1	10.9 ± 4	−2.8, 0.31	0.10
Functioning, mean ± SD				
PCS (SF-36)	213.6 ± 63.8	268.3 ± 59.7	31.5, 78.4	<0.01
MCS (SF-36)	238.8 ± 74	287 ± 67.2	21, 75.4	0.01

CS = central sensitization; SD = standard deviation; CI = confidence interval; PCS = physical component scale; MCS = mental component scale; SF-36 = 36-item short-form health survey; n = sample size.

**Table 5:** Between-group differences in anxiety, depressive symptoms, and fatigue.

Outcomes	CS Group (n = 82)	Non-CS Group (n = 41)	95% CI	*p*-Value
HADS, mean ± SD				
Anxiety	9.6 ± 3.6	7 ± 2.7	−3.7, −1.3	<0.001
Depressive symptoms	5.6 ± 3.2	4.2 ± 3.2	−2.6, −0.18	<0.001
BFS, mean ± SD				
Physical	3.8 ± 2.1	2.4 ± 1.8	−2, −0.62	0.001
Mental	3.6 ± 2.5	2.5 ± 2.3	−2, −0.14	0.02

CS = central sensitization; SD = standard deviation; CI = confidence interval; HADS = hospital anxiety and depression scale; BFS = Brugmann fatigue scale; n = sample size.

**Table 6:** Between-group differences in pressure pain thresholds.

Outcomes	CS Group (n = 82)	Non-CS Group (n = 41)	95% CI	*p*-Value
Symptomatic site, mean ± SD				
Trapezius (KPa)	3.8 ± 2.3	5 ± 2.4	0.06, 1.8	0.03
Lumbar (KPa)	5.2 ± 2.1	7.1 ± 3.3	0.08, 2.8	<0.001
Remote site, mean ± SD				
Hand (KPa)	3.8 ± 1.3	4.5 ± 1.8	0.08, 1.2	0.02
Calf (KPa)	4.5 ± 1.5	5.5 ± 2.1	0.03, 1.7	0.03

CS = central sensitization; SD = standard deviation; CI = confidence interval; KPa = kilopascal; n = sample size.

## Data Availability

Data are contained within the article.
